# Assessment of radionuclides in the soil of residential areas of the Chittagong metropolitan city, Bangladesh and evaluation of associated radiological risk

**DOI:** 10.1093/jrr/rru073

**Published:** 2014-09-18

**Authors:** Quazi Muhammad Rashed-Nizam, Md. Mashiur Rahman, Masud Kamal, Mantazul Islam Chowdhury

**Affiliations:** 1 Department of Physics, University of Chittagong, Chittagong-4331, Bangladesh; 2 Radioactivity Testing and Monitoring Laboratory, Bangladesh Atomic Energy Commission, Bangladesh; 3 Southern University, 739/A, Mehedibag Road, Chittagong, Bangladesh

**Keywords:** radionuclides, gamma ray spectrometry, BEGe detector, radiological hazard parameters, Excess of Lifetime Cancer Risk (ELCR)

## Abstract

Soil samples from the three residential hubs of Chittagong city, Bangladesh were analyzed using gamma spectrometry to estimate radiation hazard due to natural radioactive sources and anthropogenic nuclide ^137^Cs. The activity concentration of ^226^Ra was found to be in the range 11–25 Bq.kg^−1^, ^232^Th in the range 38–59 Bq.kg^−1^ and ^40^K in the range 246–414 Bq.kg^−1^. These results were used to calculate the radiological hazard parameters including Excess of Lifetime Cancer Risk (ELCR). The estimated outdoor gamma exposure rates were 40.6–63.8 nGy.h^−1^. The radiation hazard index (radium equivalent activity) ranged from 90–140 Bq.kg^−1^. The average value of the ELCR was found to be 0.21 × 10^−3^, which is lower than the world average. Sporadic fallout of ^137^Cs was observed with an average value of 2.0 Bq.kg^−1^.

## INTRODUCTION

Nuclear radiation has become a huge public concern all over the world, even though nuclear radiation is an inevitable part of our natural environment. Apart from cosmic rays, the soil of our earth is an important source of nuclear radiation. A number of natural radionuclides, namely uranium (^238^U), thorium (^232^Th) and their decay products (^226^Ra, ^212^Pb, etc.) and potassium isotope (^40^K) are observed as inherent soil contents. These natural radionuclides contribute to the radiation exposure, externally through gamma ray emission and also internally through inhalation and the food chain [1]. The use of nuclear technology also generates many long-lived radionuclides, of which ^137^Cs is the most abundant one [2]. This artificial radionuclide enters the environment largely as a result of nuclear weapon tests, accidents in nuclear power plants and the geological repository of nuclear wastes [3] and then spreads out into distant locations through atmospheric convection [4].

Knowledge of the distribution of both natural and anthropogenic radionuclides is essential for the assessment of radiation hazard. The concentration of natural radionuclides in soil is found to vary significantly from place to place [5]. Hence, surveys of terrestrial radionuclides have attracted great interest throughout the world [6–10]. In Bangladesh, there were also few studies in different regions [11, 12]. This study was conducted in three populated residential areas of Chittagong city, Bangladesh.

Chittagong is the busiest seaport city of Bangladesh. This commercial city spans 91°45′E to 91°54′E in longitude and 22°14′N to 22°24′N in latitude and the area is 168 square km. The terrain is mainly hilly; the highest point, known as Batali Hill, is 85 m above sea level. This metropolis is the second-largest populated city in Bangladesh; its population density is 15 351 per km^2^ [13].

## MATERIALS AND METHODS

### Sample collection and preparation

Topographically Chittagong city is a branch of the Himalayas [14]. The eastern border of the city is formed by the Karnaphuli River; its estuary is the southern periphery and the Bay of Bengal is on the west. Along these three boundaries, the city stands on the low plain land, but the central and northern part of the city is hilly. One of the sampling sites was Halishahar residential area which is situated beside the Bay of Bengal. Another site, Chandgaon residential area was chosen adjacent to the Karnaphuli River. The third sampling site, Nasirabad residential area was chosen from the central hilly region of the city. These sampling sites were chosen in order to find out any difference that might be present in the radionuclide contents due to different geological conditions.

**Fig. 1.**
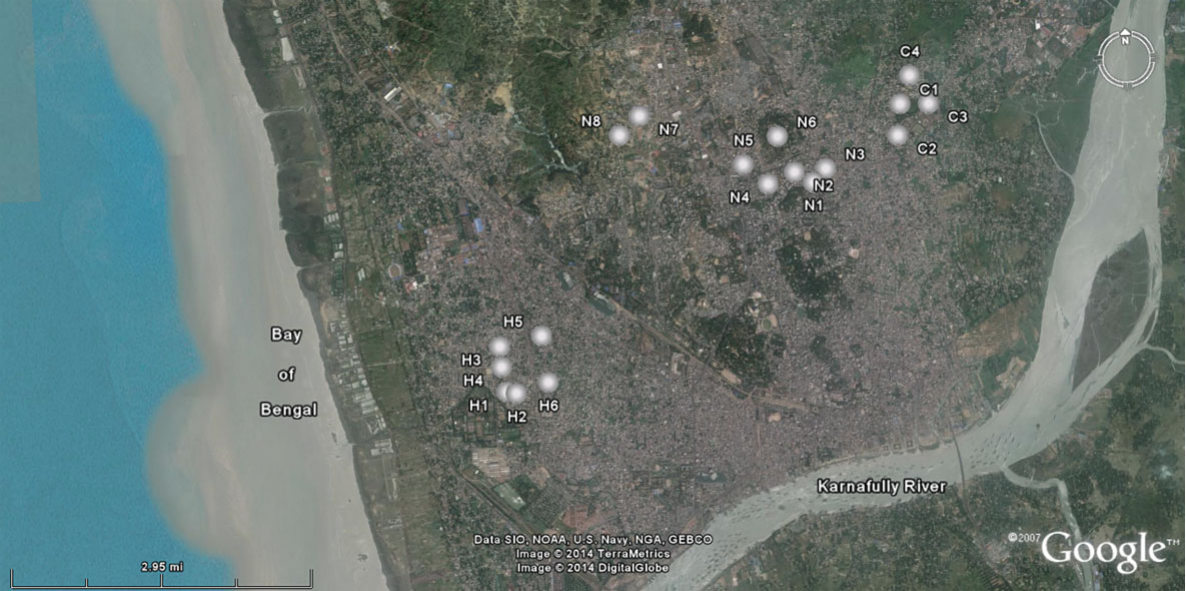
Location of sampling point.

In each of these residential hubs, densely populated places were chosen for the collection of soil samples. Locations of sampling sites were recorded using the assisted global positioning system (GPS). Figure 1 shows the location of sampling sites in Google Maps. All kinds of dirt, biological and non-biological, was swept away from the sampling site and then the surface soil was collected from an area of 15 cm × 15 cm up to a depth of 5 cm. The soil samples were prepared according to standard procedures [1]. After cleaning and drying in the sun, the soil samples were ground to fine powder. The soil samples were then dried in an electric oven at a temperature of 80°C for 24–48 h in order to evaporate off all the water content of the soil. After that, samples were kept in airtight plastic containers for a period of one month in order to bring the soil samples into a state of secular equilibrium between the long-lived parent radionuclides (^226^Ra and ^228^Ra) and their short-lived progeny.

### Analysis of soil samples

The concentration of radionuclides in the soil samples was studied by gamma spectroscopy. A Broad Energy Germanium (BEGe) detector (BE3820, made by Canberra Industries Inc., USA, www.canberra.com) was used to record the gamma emission from the soil samples. This detector can efficiently measure gamma emission in the energy range from 3 keV to 3 MeV. The measured resolution of the detector was 1.9 keV (FWHM) at a gamma energy of 1332 keV. Efficiency was measured and the calibration of the detection system was performed against the standard sources provided by International Atomic Energy Agency (IAEA). The gamma spectrum of the soil samples was analyzed using the Canberra Genie-2000 spectroscopy software.

The activity of ^226^Ra was estimated by averaging the measured activities of ^214^Pb (241.98, 295.22 and 351.93 keV lines) and ^214^Bi (609.31, 1120.29 and 1238.11 keV lines). For the estimation of the activity of ^232^Th, the measured activities of ^228^Ac (338.32, 911.20 and 968.97 keV lines), ^212^Pb (238.63 keV line), ^212^Bi (727.33 keV line) and ^208^Tl (583.19 keV line) were considered [1]. The intensities of these gamma emissions were taken from the library Nuclide-LARA [15].

### Activity concentrations and radiological hazard parameters calculation

The activity concentration of a radionuclide was determined by the unitary method [16]:

(1) $$Activity\;(Bq/kg)=\frac{c}{e*i*m}$$

where, *c* is the net count per second, *e* is the measured counting efficiency of the detector, *i* is the intensity of the gamma line from the radionuclide and *m* is the mass of the soil sample in kilograms.

Natural radionuclides ^226^Ra, ^232^Th and ^40^K in soil and sediment are observed to vary from place to place. So, for the assessment of radiation hazards associated with these radionuclides, the outdoor gamma ray exposure rate in air at one meter height above the ground due to the natural radionuclides in soils was calculated by the following formula [5]:

(2) $$D(nGy/h)=0.\text{462}A_{Ra}+0.\text{6}0\text{4}A_{Th}+0.0\text{417}A_{K}$$

where, *A_Ra_, A_Th_* and *A_K_* are the average activity concentrations of ^226^Ra, ^232^Th and ^40^K, respectively, in soils in units of Bq.kg^−1^. Due to the outdoor exposure *D*, the average annual effective dose (*H*) to adults was estimated on the assumption that the outdoor occupancy fraction is 0.2 and the Gray to Sievert transformation factor is 0.7 Sv/Gy:

(3) $$\;H(\text{mSv})=D(nGy/h)\times \text{876}0\;h\times 0.\text{2}\times 0.\text{7 Sv}/\text{Gy}\times \text{1}0^{-6}$$

In order to compare the combined radiological effect due to the natural radionuclides, it is now common practice to calculate the radium equivalent activity (*Ra_eq_*) and representative level index (*I_γr_)* using the following equation [17, 18]:

(4) $${Ra}_{eq}=A_{Ra}+\text{1}.\text{43}A_{Th}+0.0\text{77}A_{K}$$

(5) $$\text{and}\;I_{\gamma r}=0.0\text{1}A_{Ra}+0.0\text{1 }A_{Th}+\text{7}x\text{1}0^{-4}A_{K}$$

where, *A_Ra_*, *A_Th_*, and *A_K_* are the specific activities of ^226^Ra, ^232^Th and ^40^K in Bq.kg^−1^, respectively, assuming that ^137^Cs can be neglected as it contributes very little to the total dose from the environmental background [19–21].

Since gamma radiation provides information on the Excess of Lifetime Cancer Risks (ELCRs), it is necessary to measure this parameter. The ELCR was calculated by using the following equation [7]:

(6) $$ELCR=H_{eff}*DL*RF$$

where, *DL* is the duration of life (70 years for Bangladeshi people) and RF is the risk factor (Sv^−1^). For stochastic effects, ICRP 60 recommends *RF* = 0.05 for the public exposure [22].

## RESULTS

### Specific activities of ^226^Ra, ^232^Th and ^40^K

The measured activity concentration of natural radionuclides ^226^Ra, ^232^Th and ^40^K in the soil samples are listed in Table 1. The activity concentration of ^226^Ra was found to be in the range of 11 ± 1.6 Bq.kg^−1^ to 25 ± 2.4 Bq.kg^−1^; ^232^Th spanned from 38 ± 2.3 Bq.kg^−1^ to 59 ± 4.2 Bq.kg^−1^ and ^40^Ka ranged from 246 ± 30 Bq.kg^−1^ to 414 ± 40 Bq.kg^−1^. The concentrations of natural radionuclides in the soils of the Chittagong residential area were observed not to vary greatly, as observed in the different regions of the world listed in the Table 2.

**Table 1.** Activity concentrations of ^226^Ra, ^232^Th, ^40^K and ^137^Cs in soil samples

Name of the location	Location	Sample ID	Activity concentration in Bq.kg^−1^			
			^226^Ra	^232^Th	^40^K	^137^Cs
Chandgaon	22°22′34.24″N 91°50′47.82″E	C1	15 ± 1.4	46 ± 2.2	255 ± 31	1.4 ± 0.3
	22°22′22.29″N 91°50′49.54″E	C2	20 ± 4.0	47 ± 2.1	295 ± 36	2.8 ± 0.5
	22°22′34.24″N 91°50′47.82″E	C3	16 ± 1.4	47 ± 2.5	340 ± 31	ND
	22°22′37.01″N 91°50′53.69″E	C4	16 ± 1.4	37 ± 2.3	268 ± 31	ND
Halishahar	22°20′17.78″N 91°47′03.52″E	H1	12 ± 1.1	42 ± 1.1	278 ± 28	ND
	22°20′17.70″N 91°47′01.52″E	H2	18 ± 1.4	42 ± 3.2	312 ± 30	ND
	22°20′40.36″N 91°47′00.91″E	H3	25 ± 1.8	44 ± 2.5	315 ± 31	ND
	22°20′44.59″N 91°46′53.50″E	H4	24 ± 1.7	51 ± 4.3	414 ± 40	ND
	22°20′44.93″N 91°47′23.86″E	H5	18 ± 1.5	47 ± 3.3	368 ± 29	ND
	22°20′17.78″N 91°47′03.52″E	H6	11 ± 1.6	40 ± 3.3	312 ± 27	ND
Nasirabad	22°21′56.37″N 91°49′57.53″E	N1	25 ± 2.4	59 ± 4.2	406 ± 35	ND
	22°22′02.55″N 91°49′47.65″E	N2	17 ± 2.3	45 ± 2.4	359 ± 36	1.2 ± 0.3
	22°21′56.86″N 91°49′59.64″E	N3	14 ± 1.3	39 ± 2.3	246 ± 30	ND
	22°22′03.15″N 91°49′41.09″E	N4	19 ± 1.5	43 ± 3.3	275 ± 31	ND
	22°22′07.91″N 91°49′35.77″E	N5	22 ± 2.2	45 ± 2.2	299 ± 30	ND
	22°22′17.45″N 91°49′38.70″E	N6	17 ± 2.0	41 ± 2.8	277 ± 31	ND
	22°22′34.73″N 91°48′23.47″E	N7	17 ± 2.4	55 ± 3.5	376 ± 29	ND
	22°22′25.86″N 91°48′11.69″E	N8	23 ± 3.0	49 ± 4.0	377 ± 33	ND
		Average	18 ± 4.2	46 ± 5.5	321 ± 52	2.0 ± 0.9

**Table 2.** Activity concentrations in different countries [6, 8–10, 26, 27–38]

Country	^226^Ra	^232^Th	^40^K	^137^Cs
Algeria	–	2–144	35–1405	0.1–43
Belgium	5–50	5–50	70–900	–
Bulgaria	9–77	5–110	11–760	–
China	2–440	33–88	442–913	–
Cairo, Egypt	5.3–66.8	5–37.3	41.5–418	0–35.7
Denmark	9–29	8–30	240–610	–
France	38 (9–62)	38 (16–55)	599 (120–1026)	–
Greece	1–240	43 (1–190)	1130 (12–1570)	1.8–11.1
Hong Kong SAR	20–110	16–200	80–1100	–
India	7–81	14–160	38–760	≤1–2.88
Italy	17–630	16–62	398–649	
Iran	8–55	5–42	250–980	–
Jordan	16.3–7.3	7.6–16.2	121.8–244.8	1.9–5.3
Japan	6–98	2–88	15–990	–
Kuwait	–	6	227	–
Luxembourg	6–52	7–70	80–1800	–
Netherlands	–	22–77	290–700	–
Norway	720–1760	26–50	700–1400	–
Pakistan	–	22–59	303–945	1–5
Poland	5–120	4–77	110–970	–
Portugal	8–65	22–100	220–1230	–
Romania	8–60	11–75	250–1100	–
Spain	6–250	2–210	25–1650	10–60
Switzerland	10–900	4–70	40–1000	–
Taiwan	44.7–10.6	12.2–44.2	195.3–640	0–12.1
Turkey	10–58	8–91	117–1204	2–81
USA (Lousiana)	64 (34–95)	36 (4–130)	472 (43–719)	5–58
Bangladesh (Chittagong)	18 (10.58–24.60)	46 (37.56–58.80)	321 (245.9–414.1)	2.00 (1.2–2.8)

**Fig. 2.**
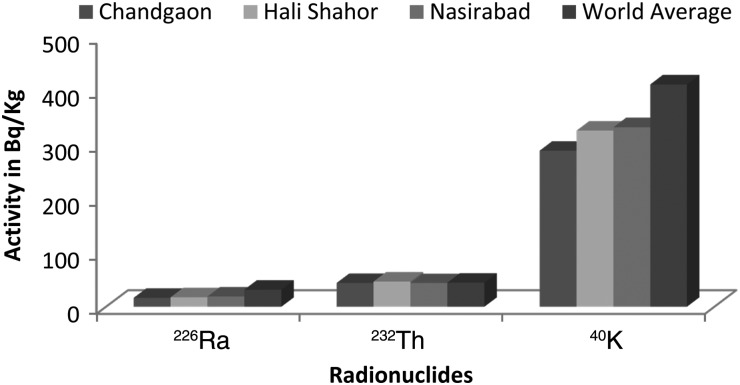
The average activity of natural radionuclides in the three residential hubs in comparison with the world average values.

**Fig. 3.**
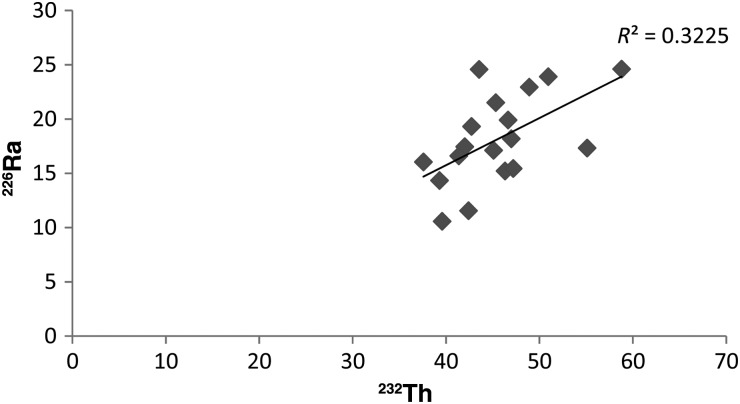
Correlation between **^226^**Ra and **^232^**Th radionuclides.

These three residential areas give the impression of being geographically different, but the average activity concentrations in the soils were found to be very similar, as shown in Fig. 2. Also, none of these terrestrial radionuclides exceeded the world average value [5]. Figure 2 illustrates the average activity concentration of natural radionuclides in the three residential hubs in comparison with the world average values [5]. Table 3 shows the concentration of terrestrial radionuclides in the sediments of the Bay of Bengal [4] and the Karnaphuli River [23]. In comparison with these tabulated values, the soil samples of the residential areas contain smaller amounts of natural radionuclides. It was found that the sediment of the Bay of Bengal exhibits a strong correlation (R^2^ = 0.97) [23] between ^226^Ra and ^232^Th radionuclides. As shown in Fig. 3, no such strong correlation (R^2^ = 0.32) was observed in the soil samples of the Chittagong city area. Hence the soil of Chittagong city is radiologically different from the nearby river and bay.

**Table 3.** Concentrations of terrestrial radionuclides in the sediments of the Karnaphuli River and the Bay of Bengal [4, 23]

Radionuclides	Range of Activity concentration (Bq/kg) (Average)		
	Karnaphuli River	Bay of Bengal	Chittagong Residential Area
^226^Ra	19–85 (35.9)	15–47 (30.9)	10.58–58.80 (18)
^232^Th	51–88 (65.5)	29–95 (61.7)	37.56–58.80 (46)
^40^K	217–320 (272.0)	143–1093 (467.8)	245.9–414.1 (321)

### Specific activities of anthropogenic radionuclides ^137^Cs

In Chittagong city, there is no anthropogenic nuclear activity except the use of ^60^Co and other short-lived radionuclides for medical purposes. Moreover, there is no history of accidents at the only research reactor located in the capital city Dhaka. However, due to atmospheric fall-out, the nuclear fission product ^137^Cs has been observed in a number of locations but not everywhere in Bangladesh. The same pattern was also observed in our study. ^137^Cs was found not in all the soil samples, and the maximum value was 1.3 ± 0.2 Bq.kg^−1^. In Bangladesh, the maximum allowable limits of this radionuclide in dairy and non-dairy foodstuffs are 95 Bq.kg^−1^ and 50 Bq.kg^−1^, respectively [24]. So, it can be asserted that the observed ^137^Cs would not cause contamination of the foodstuffs at a level of concern for radiation risk.

### Radiological hazard parameters

Due to the natural radionuclides in the soils, the outdoor absorbed dose rate was found to be in the range of 40.6–63.8 nGy.h^−1^ with an average of 49.3 ± 3.9 nGy.h^−1^. Except for the highest value 63.8 nGy.h^−1^, observed at the sample N1 (Nasirabad), none of the other values was above the world average value of 58 nGy.h^−1^. The annual effective dose was estimated to be in the range of 0.050–0.078 mSv, with an average of 0.060 ± 0.005 mSv; this average value is lower than the danger limit of 0.07 mSv per year. The radium equivalent activity was found to be in the range of 89.5–139.9 Bq.kg^−1^ with an average of 108.0 ± 8.5 Bq.kg^−1^, which is also less than the maximum limit of 370 Bq.kg^−1^ recommended by the OECD [17]. The resulting average of the representative level index (I_γr_) was 0.79 Bq.kg^−1^ with ranges from 0.65–1.02 Bq.kg^−1^, which is greater than the world average value of 0.66 Bq.kg^−1^ [25]. From Table 4, we see that the value of the ELCRs ranges from (0.17–0.27) × 10^−3^ with the average value of 0.21 × 10^−3^, which is lower than the world average value of 0.25 × 10^−3^ [26]. Thus the background nuclear radiation in Chittagong city is within the accepted value. The values of the radiological hazard parameters for each sample are given in Table 4.

**Table 4.** The radiological hazard indices in the three residential hubs of Chittagong city

Location	D nGy.h^−1^	H mSv.a^−1^	Ra_eq_ Bq.kg^−1^	I_γr_ Bq.kg^−1^	ELCR × 10^−3^
Chandgaon	45.65 ± 3.26	0.056	101.1 ± 6.9	0.73	0.20
	49.68 ± 4.66	0.061	109.3 ± 9.87	0.80	0.21
	49.83 ± 3.45	0.061	109.1 ± 7.35	0.80	0.21
	41.27 ± 3.34	0.051	90.4 ± 7.1	0.66	0.18
Average	46.61 ± 4.05	0.06	102.41 ± 8.01	0.75	0.20
Halishahar	42.52 ± 2.34	0.052	93.6 ± 4.84	0.69	0.18
	46.44 ± 3.88	0.057	101.5 ± 8.3	0.74	0.20
	50.77 ± 3.62	0.062	111.1 ± 7.71	0.81	0.22
	59.08 ± 5.05	0.072	128.6 ± 10.91	0.94	0.25
	52.12 ± 3.87	0.064	113.7 ± 8.40	0.84	0.22
	41.79 ± 3.85	0.051	91.2 ± 8.38	0.67	0.18
Average	48.79 ± 6.55	0.06	106.61 ± 14.77	0.78	0.21
Nasirabad	63.81 ± 5.13	0.078	139.9 ± 11.16	1.02	0.27
	50.10 ± 4.02	0.061	109.2 ± 8.5	0.80	0.22
	40.61 ± 3.31	0.05	89.5 ± 7.04	0.65	0.17
	46.17 ± 4.01	0.057	101.5 ± 8.68	0.74	0.20
	49.76 ± 3.56	0.061	109.3 ± 7.58	0.80	0.21
	44.23 ± 3.94	0.054	97.1 ± 8.46	0.71	0.19
	56.97 ± 4.4	0.07	125.1 ± 9.56	0.92	0.24
	55.87 ± 5.14	0.069	121.9 ± 11.17	0.89	0.24
Average	50.94 ± 7.59	0.06	111.7 ± 16.49	0.79	0.21
World average	58	0.07	129.1^†^	0.66	0.25

^†^This is calculated on the basis of world average values of ^226^Ra, ^232^Th and ^40^K.

**Fig. 4.**
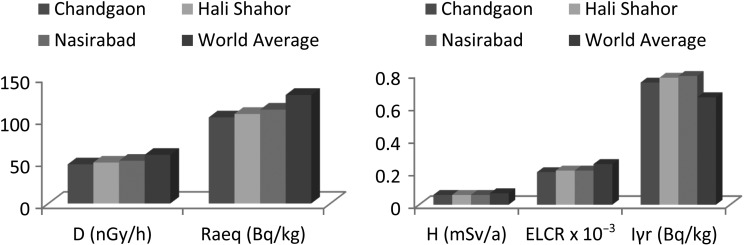
Comparison of radiological hazard indices with world average.

## DISCUSSION

The distribution of terrestrial radionuclides in three residential hubs—Chandgaon, Halishahar and Nasirabad—of the Chittagong metropolitan city, Bangladesh was measured using gamma spectrometry. Nasirabad is a hilly area but the other two regions consist of low plain land. No significant difference in the concentration of natural radionuclides was observed in the soils of these three areas, as shown in Table 1 and Fig. 2. Hence the radiological content of the soil was not found to depend on the nature of landscape, i.e. whether hilly or plain land. The uniform nature of the soils from a radiological point of view implies that the soils in these areas were formed through the same geological processes.

The average activity concentration of natural radionuclides ^226^Ra, ^232^Th and ^40^K in the soil were found to be 18.2 ± 1.9 Bq.kg^−1^, 45.5 ± 2.9 Bq.kg^−1^ and 320.6 ± 31.6 Bq.kg^−1^, respectively; these measured values are within the world average values (32 Bq.kg^−1^ for ^226^Ra, 45 Bq.kg^−1^ for ^232^Th and 412 Bq.kg^−1^ for ^40^K) [5]. Again, these average values and also the distributions were found to vary considerably from that observed in the sediments of the Bay of Bengal and the Karnaphuli River (Table 2) which surround the city. The distributions of radionuclides in the soils of Chittagong residential areas are different from that of the other regions of the country, as shown in Table 5 and the concentrations of radionuclides are lower than that in other countries (Table 2).

**Table 5.** The activity concentrations of radionuclides in soil of the different regions of Bangladesh [23, 39, 40]

District	Activity in Bq.kg^−1^			
	^226^Ra	^232^Th	^40^K	^137^Cs
Barishal	51 ± 3	60 ± 5	670 ± 23	ND
Pirojpur	42 ± 3	97 ± 7	1701 ± 35	ND
Jhalokati	43 ± 2	77 ± 6	720 ± 27	1.0
Patuakhali	36 ± 2	52 ± 5	549 ± 24	ND
Barguna	38 ± 2	64 ± 6	739 ± 24	ND
Madaripur	25 ± 2	61 ± 6	656 ± 22	ND
Khulna	44 ± 3	62 ± 6	811 ± 30	10
Shatkhira	44 ± 3	92 ± 8	1762 ± 38	13
Jessore	44 ± 4	77 ± 7	602 ± 25	ND
Bhola	17 ± 2	33 ± 3	744 ± 22	ND
Chittagong Ship Breaking Area	31 ± 3	62 ± 5	468 ± 31	ND
Chittagong Residential Area	18 ± 4.2	46 ± 5.5	321 ± 52	2 ± 0.9

As observed in a number of areas of Bangladesh, the anthropogenic radionuclide ^137^Cs was found in few places as a result of atmospheric fallout, but the concentrations of the ^137^Cs were too low to cause any serious health concern. Again, the radiological hazard indices, as shown in Fig. 4 indicate that, the natural nuclear radiation in the city is well below the habitable limit. Average value of ELCR indicates no cancer risk in the Chittagong city due to terrestrial nuclear radiation. Supplementary research on the relation between ELCR and mortality [7] is necessary for the assessment of risk based on ELCR.
